# Long-term impacts of COVID-19 on systemic inflammation and control of breathing reflexes: an observational cohort study

**DOI:** 10.1186/s12931-025-03473-6

**Published:** 2026-01-21

**Authors:** Veronica L. Penuelas, Kathy Pham, Shyleen Frost, Indira S. Harahap-Carrillo, Abel Vargas, Kristina V. Bergersen, Yuxin He, Meera G. Nair, Marcus Kaul, Erica C. Heinrich

**Affiliations:** Division of Biomedical Sciences, School of Medicine, University of California, Riverside, CA USA

**Keywords:** COVID-19, Chemoreflex, Systemic inflammation, Control of breathing, Hypoxia, Hypercapnia, Ventilatory response, Inflammatory biomarker

## Abstract

**Background:**

The COVID-19 pandemic resulted in over 7 million reported deaths and over 700.4 million reported infections to-date. Many individuals who recover from COVID-19 report prolonged dyspnea, sometimes persisting for months. Furthermore, COVID-19 has been linked to systemic and neuronal inflammation which may have downstream impacts on the neural control of breathing. Therefore, we hypothesized that individuals recovered from COVID-19 may exhibit changes in their ventilatory chemosensitivity to carbon dioxide and hypoxia, and that these changes may be linked to systemic inflammation.

**Methods:**

To test this hypothesis, we measured baseline ventilatory patterns and chemoreflex sensitivity in individuals recovered from COVID-19 (*n =* 77) and individuals with no prior COVID-19 infection (*n =* 41). Peripheral venous blood samples were also collected for inflammatory biomarker expression and profiling.

**Results:**

Recovered participants demonstrated a small but progressive decrease in the hypercapnic ventilatory response under a co-stimulus with hypoxia (control vs. 24-month post-recovery; *p =* 0.023). Additionally, we identified several significant correlations between plasma inflammatory markers and ventilatory chemoreflex characteristics, including a positive correlation between SAA and CRP and the ventilatory response to hypoxia (*p <* 0.05 within recovered and control cohorts). Finally, expression of six vascular inflammatory markers (Myoglobin, NGAL, MMP-2, OPN, IGFBP-4, and Cystatin C) was unexpectedly decreased in recovered participants compared to the control cohort for up to one-year post recovery.

**Conclusions:**

Overall, this data indicates that COVID-19 and other acute viral infections may have a modest impact on the chemoreflex control of breathing as well as systemic inflammatory profiles, and that these changes may be linked to each other. These findings may strengthen our understanding of the pathology of long-COVID symptoms.

**Supplementary Information:**

The online version contains supplementary material available at 10.1186/s12931-025-03473-6.

## Background

COVID-19 (Coronavirus Disease) caused more than 700.4 million reported infections and seven million deaths worldwide, and millions of hospitalizations in the United States, since its emergence in late 2019. The infection is associated with a large systemic inflammatory response, or cytokine storm, as well as impaired lung function in individuals with moderate to severe disease [1, 2]. Among unique COVID-19 symptoms was the presence of “silent hypoxemia”, which described significant hypoxemia without substantial dyspnea or respiratory distress [3]. In addition to silent hypoxemia, other respiratory symptoms including prolonged dyspnea [4] and sleep disordered breathing following recovery [5], implicated potential changes in the chemoreflex control of breathing in the pathology of COVID-19.

The hypoxic and hypercapnic ventilatory responses (HVR and HCVR, respectively), or the reflex increases in breathing in response to hypoxia or hypercapnia, as well as baseline respiratory drive, are highly variable both within and across individuals [6]. This plasticity is observed in physiological and pathological conditions including acclimatization to sustained hypoxia [7, 8], obstructive and central sleep apnea [9–11], and heart failure [12]. The molecular and neurophysiological mechanisms underlying plasticity in these chemoreflexes has been an area of much study [6]. This prior work indicates that local tissue and neuroinflammatory signals play a role in ventilatory acclimatization to hypoxia and long-term facilitation of ventilatory chemoreflexes [13]. However, whether or not systemic inflammation may be communicated to the respiratory centers and play a role in sensitizing or blunting these reflexes remains unclear [14].

Several studies have examined the impact of systemic inflammation on breathing control in animal models and humans over different timescales of exposure. Bavis et al. found that rats exposed to chronic hypoxia from birth developed a blunting of the HVR, which was associated with increased expression of proinflammatory cytokines interleukin-6 (IL-6), interleukin-1beta (IL-1β), and tumor necrosis factor alpha (TNF-α) [14]. Additionally, Popa et al. determined that ibuprofen treatment was sufficient to block the acute increase in HVR expected in response to hypoxia exposure in rats [15]. Similarly, blunted HVRs were also observed after acclimatization to high altitude in humans when treated with ibuprofen every 4 hours [16]. This data demonstrates a potential role of systemic inflammation in modulating ventilatory chemoreflexes. Acute inflammatory signaling is necessary to initiate increases in the HVR, such as during ventilatory acclimatization to high altitude, but chronic sustained inflammation may blunt the HVR over longer time periods. These mechanisms may underly changes in ventilatory control as a result of COVID-19 or other conditions associated with substantial systemic or pulmonary inflammation. Further supporting a potential impact of COVID-19 on respiratory control, autopsy evidence from COVID-19 patients revealed potential direct infiltration of the virus into the brainstem, as well as increased presence of inflammatory factors in this region, which may also directly impact brainstem respiratory center function [17, 18].

Based on this data and reported long-COVID respiratory and sleep symptoms, we hypothesized that levels of systemic inflammation in recovered patients would be associated with changes in the ventilatory chemoreflex control of breathing, such as blunting of the HVR following a prolonged period of systemic inflammation. To test this, we measured the HVR, HCVR, and peripheral blood inflammatory marker expression in individuals recovered from COVID-19 over a range of timescales, as well as in individuals that had never tested positive or been symptomatic for COVID-19.

## Methods

### Ethical approval

This study was approved by the University of California (UC) Riverside Institutional Review Board (HS-20–128) and performed in accordance with the *Declaration of Helsinki*, except for registration in a database. Consent procedures were performed in the participant’s native language with study personnel fluent in the language (English or Spanish). All participants were provided with a copy of the consent form prior to their appointment and were informed of the purpose of the study, including all risks and benefits. After all information was provided, both written and verbal consent were required to move forward with the study.

### Participant demographics and inclusion criteria

We recruited 118 participants for a cross-sectional study between April of 2022 and May of 2023. Recruitment was performed via word of mouth, social media, and flyers around the UC Riverside campus and wider Riverside, California area. Of the 118 participants, 41 reported never having COVID-19 as evidenced by no history of a positive COVID-19 test or any related symptoms (control group) and 77 reported confirmed positive cases of COVID-19 (recovered group), indicated by a positive test or medical treatment for COVID-like symptoms. A time-post-recovery analysis was conducted among the recovered group, with each recovered participant tested only once. Recovered participants were separated into groups at 3-month intervals for the first year following recovery; however, two of the 3-month groupings (6 to 9 months and 9 to 12 months) were merged into a 6-month grouping due to low participant numbers. Of the recovered participants, five were previous control participants that returned after COVID-19 infection, making it possible to investigate within-subject changes in ventilatory control before and after COVID-19 infection in this small subsample. Each participant self-reported their biological sex at birth, gender identity, and ethnicity (Table 1). Two participants did not currently identify as male or female gender at the time of the study but were both assigned male sex at birth and were included in the male category for data analysis purposes. Neither individual reported the current use of any hormone treatment therapy.

**Table 1:** Participant demographics

	Male	Female
Control (*n =* 41)	20	21
Recovered (*n =* 77)	29	48
Total (*n =* 118)	49 (41.52%)	69 (58.47%)

Participants were asked not to consume caffeine the morning of the study and to abstain from taking anti-inflammatory medication, corticosteroids, or other medications that could potentially interfere with control of breathing measures and inflammatory marker expression [13] for at least 12 h prior to testing. If participants were not able to stop taking the referenced medications, their data was excluded. Of note, participants were not required to fast prior to their study appointments since, in a related study, blood sample measurements were compared to intensive care unit (ICU) patient samples, for which it was not possible to require fasting [19].

Inclusion criteria included age ≥ 18 years. Exclusion criteria included pregnancy due to links between hypoxia exposure and development of preeclampsia [20]. Participants with current severe cardiac or pulmonary illness were excluded or conducted limited testing within safe limits. One participant with a history of lung cancer and partial lobectomy completed only an abbreviated version of the chemoreflex test and did not participate in the hypoxia phase. Exclusion criteria also included confirmed or suspected active COVID-19 infection.

### Study design

Prior to arriving for their study appointment, participants were required to complete a screening survey to verify they had no current COVID-19 infection or other illness. Following informed consent procedures, basic physiological measures were collected. Blood pressure was taken using a stethoscope (Littmann, St. Paul, MN) and manual sphygmomanometer (Elite Medical Instruments, Orange County, CA), body temperature was collected from the forehead using an infrared thermometer (Femometer, Princeton, NJ) and height and weight were recorded. Participants then completed a series of questionnaires regarding their past medical history, demographics, and long-COVID symptoms [21]. Peripheral venous blood samples were then collected via standard venipuncture procedures by a licensed phlebotomist. Participants then completed a spirometry test, used to measure forced expired volume in 1 s (FEV_1_) and forced vital capacity (FVC), ensuring at least two high quality recordings with stable measures. During this test, participants were instructed to take a deep breath and exhale as fast and as forcefully as possible into a mouthpiece connected to a respiratory air filter and flowmeter (AD Instruments). This test was followed by ventilatory chemoreflex measures. The complete study appointment lasted approximately two hours.

### Blood sample processing

20 mL of blood was collected in two vacutainer tubes containing EDTA. Samples were kept at room temperature and processed within one hour. Tubes were centrifuged at 2500× g for ten minutes. After separation, plasma used for inflammatory cytokine assays was stored at −20 °C for short term storage and at −80 °C for long term storage.

### LEGENDPlex assays

Plasma samples were chosen at random from the larger sample set for cytokine biomarker analyses. Plasma inflammatory marker expression was measured via LEGENDplex™ multiplex immunoassays (BioLegend, San Diego, CA, US). We utilized the Human Anti-Virus Response Panel (control: *N =* 41; and recovered: *N =* 72; IL-1β, IL-6, CXCL8 (IL-8), IL-10, IL-12p70, IFN-α2, IFN-β, IFN-λ1 (IL-29), IFN-λ2/3 (IL-28a/b), IFN-γ, TNF-α, CXCL10 (IP-10), and GM-CSF), and Human Vascular Inflammation Panel 1-TC (control: *N =* 38; and recovered: *N =* 25; Myoglobin, Calprotectin (MRP8/14), Lipocalin (NGAL), C-Reactive Protein (CRP), MMP-2, Osteopontin (OPN), Myeloperoxidase (MPO), Serum Amyloid A (SAA), IGFBP-4, ICAM-1 (CD54), VCAM-1 (CD106), MMP-9, and Cystatin C), following manufacturer protocols. Each sample was treated with Triton X at a 1% concentration, diluted with assay buffer at a 1:2 ratio for the anti-virus response panel and 1:100 for the vascular inflammation panel. Samples were then tested in duplicates. Panels were read using a Novocyte Quanteon flow cytometer (Agilent Technologies, Santa Clara, CA) and expression was quantified using the LEGENDplex™ Data Analysis Software Suite from Qognit by BioLegend®. The auto-generated gating strategy was manually modified to adjust for an analyte that was not detected automatically.

### Ventilatory chemoreflex testing

Ventilatory chemoreflex measures were conducted using the modified Duffin rebreathing technique [22], as described previously [23], with the exception of a shortened hyperventilation phase [24]. Oxygen (O_2_) and carbon dioxide (CO_2_) gas analyzers, flow meters, and pulse oximeters were calibrated following manufacturer instructions within one hour prior to testing. Participants were seated and reclined in a semi-recumbent position with their legs bent and uncrossed. They were then fitted with a pulse oximeter and a 3-lead electrocardiogram (ECG). If participants were wearing nail polish, they were asked to remove it if O_2_ saturation was < 95% [25].

Participants were fitted with a vinyl mouthpiece (VacuMed, Ventura, CA, USA) attached to a rebreathing system, as described in Frost et al. [23]. A disposable respiratory filter (MLA304, AD Instruments, Colorado Springs, CO, USA) and flow meter (ML 1000, AD Instruments) were placed downstream of a silicone mouthpiece. A three-way T-valve (Hans Rudolph Inc., catalog # NC2120910, Shawnee, KS) was then fitted to allow redirection of airflow from room air to the rebreathing bag. O_2_ and CO_2_ were subsampled near the mouth at a rate of 200 mL/min by an electromagnetic O_2_ analyzer (VacuMed, model #17,625) and infrared CO_2_ analyzer (VacuMed, model #17,630, Ventura, CA). At the base of the rebreathing bag, a line for an O_2_ concentrator (DeVilbiss, Port Washington, NY) was attached to allow manual addition of O_2_ to maintain hyperoxic or hypoxic inspired partial pressure of oxygen (PO_2_) levels constant throughout each test. Nose clips (Sklar, West Chester, PA) were placed on each participant to ensure air was not being inhaled or exhaled through the nasal passageways.

During testing, participants were first instructed to relax and breathe normally for five minutes. Throughout this time, the participants were breathing room air and were asked to refrain from talking, moving, or looking at their cell phones or other devices. At the conclusion of the five-minute rest phase, the participants were asked to voluntarily hyperventilate by inhaling and exhaling slowly and deeply, avoiding panting [24]. The hyperventilation phase lasted approximately two minutes, or until the end-tidal partial pressure of carbon dioxide (P_ET_CO_2_) reached 22 mmHg. The purpose of this phase is to reduce the P_ET_CO_2_ below the ventilatory recruitment threshold (VRT) to ensure this parameter is detected during the test. This phase also ensures that when the participant begins breathing from the rebreathing bag, their alveolar gas equilibrates with the gas pressures in the bag and minimizes arteriovenous PCO_2_ differences to avoid effects of cerebral blood flow changes [22]. This equilibration is detected by a plateau in P_ET_CO_2_ shortly after the onset of rebreathing.

**Fig. 1:**
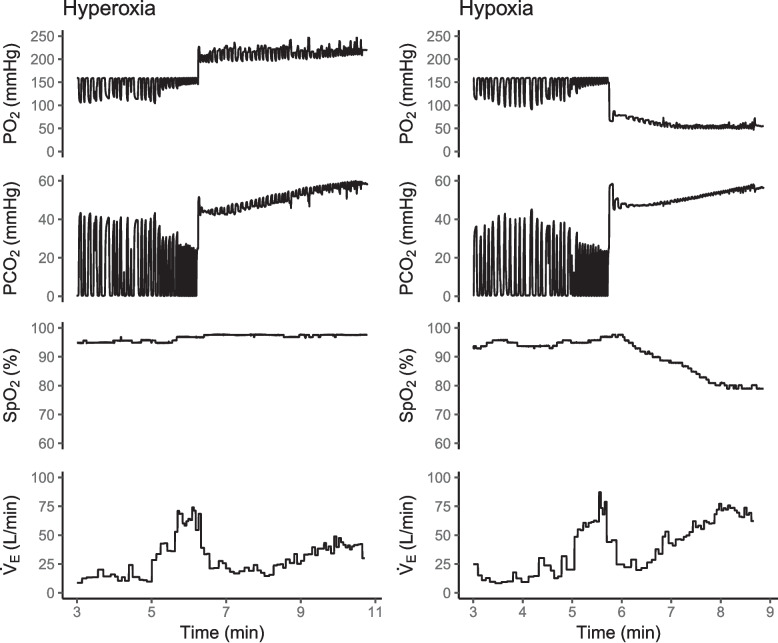
Representative raw data traces from rebreathing chemoreflex tests. Raw data for minute ventilation (V̇_E_), pulse oximetry (SpO_2_), as well as partial pressures of oxygen (PO_2_) and carbon dioxide (PCO_2_) measured at the mouth throughout two rebreathing tests in hyperoxic (target 30% inspired O_2_) (left) and hypoxic (target end-tidal PCO_2_ of 50 mmHg) (right) conditions. Recordings were taken from the same individual

Immediately after the hyperventilation phase, participants were switched from breathing room air to breathing from a 6 L bag, instructed to take two large breaths, then asked to relax. Participants stayed on the rebreathing bag for several minutes while P_ET_CO_2_ was allowed to slowly increase over time from their starting value to 60 mmHg. This typically took 8–10 min. During this time, PO_2_ in the bag was maintained at a constant level by manual addition of O_2_ from the oxygen concentrator. The test was repeated twice, under hyperoxic followed by hypoxic conditions to avoid impacts of long-term facilitation and progressive augmentation, in which baseline minute ventilation as well as the hypoxic component of the ventilatory chemoreflex is sensitive to successive episodes of hypoxia stimulus [6]. The hyperoxic gas mixture maintained an inspired oxygen concentration of 30%, and the hypoxic gas mixture maintained a P_ET_O_2_ level of 50 mmHg (P_I_O_2_ approximately 70 mmHg, allowing average desaturation to approximately 80–85%). Between these two tests, participants rested and breathed room air for 15 min to allow them to return to baseline. Tests were terminated if P_ET_CO_2_ reached 60 mmHg, peripheral oxygen saturation (SpO_2_) approached 70%, minute ventilation (V̇_E_) reached 100 L/min, or if the participant voluntarily ended the test. A raw data trace demonstrating this procedure is provided in Fig. 1.

### Data collection

During the chemoreflex test, analog output from each data source (gas analyzers, flow meter, pulse oximeter, ECG) was collected by a PowerLab data acquisition system (AD Instruments) which converted data to a digital signal that was sent to a computer for collection in LabChart 8 software (AD Instruments). The integral of the flow channel was used to record inspiratory volumes. All volumes were converted to BTPS (body temperature and pressure, saturated) units. One minute of pre-test data at the end of the rest period was used to determine resting breathing parameters.

### Chemoreflex data analysis

**Fig. 2:**
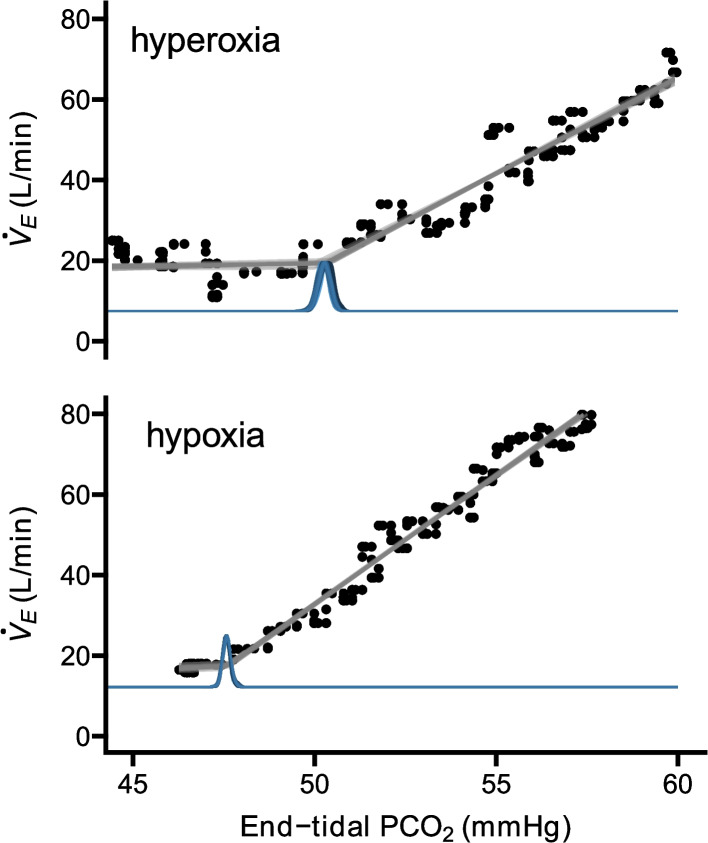
Rebreathing chemoreflex plots. Minute ventilation (V̇_E_) plotted as a function of end-tidal PCO_2_ under hyperoxic (target 30% inspired O_2_) (top) and hypoxic (target end-tidal PCO_2_ of 50 mmHg) (bottom) test conditions in the same individual. The results of the estimated VRT point via mcp bootstrap analyses are included as blue density plots on the x axis. Individual data points represent mean minute ventilation subsampled from a running average channel at about 10 samples per minute

Chemoreflex data was pre-processed in LabChart 8 and analyzed in Rstudio. The VRT in each test condition was determined using the *mcp* package in R [26], which uses Bayesian inference to identify the ideal break point in a two-slope line of best fit. The estimates of the *mcp* function were plotted alongside the raw ventilation data to visually validate the VRT estimate (Fig. 2). The HCVR slope was determined as the slope of the second segment of the “hockey stick” shaped ventilatory response curve. Two VRT and HCVR values were calculated for each participant, representing values under two distinct oxygen tensions (VRT_hyperoxia_, VRT_hypoxia_, HCVR_hyperoxia_, HCVR_hypoxia_).

Because the rebreathing technique provides ventilation rates as a function of continuously increasing P_ET_CO_2_ at two different PO_2_ levels, it allows the HVR to be calculated at any P_ET_CO_2_. We therefore calculated the HVR at three P_ET_CO_2_ levels (50 mmHg, 55 mmHg, and 3 mmHg above the VRT_hyperoxia_). These levels were chosen to ensure that HVR measures were collected above the VRT. The HVR at a given P_ET_CO_2_ was calculated as follows: The SpO_2_ was recorded at the exact time when the target P_ET_CO_2_ was reached (SpO_2*hypoxia*_ and SpO_2*hyperoxia*_). Then, to determine the ventilation rate at each of these corresponding SpO_2_ levels (V̇_E,*hypoxia*_ and V̇_E,*hyperoxia*_), the equation for the linear HCVR response curve was used. The HVR at a given ETPCO_2_ was then calculated as:

$$HVR\;=\;\frac{{}_{V_{E-\;hypoxia}-\;V_{E-hyperoxia}}}{{SpO}_{2-\;hyperoxia}-SpO_{2-\;hypoxia}}$$

For downstream analyses, chemoreflex data was quality checked by verifying the presence of abnormalities such as background noise, sensor failures or ventilatory abnormalities (i.e., coughing), insufficient data due to participants rapidly approaching 60 mmHg endpoints, or voluntarily exiting the test. This left us with 235 high quality HVR measures (*n =* 93 for HVR_50_, *n =* 69 for HVR_55_, *n =* 73 for HVR_vrt+3_). Furthermore, a coefficient of variation (CV) calculation was performed on all chemoreflex measures.

### Statistical analysis

All statistical analyses were conducted in R (Version 4.3.1). To determine if demographic variables differed across control and recovered groups, chi-squared (sex) and unpaired t-tests were utilized. To determine if there were significant effects of group (control *versus* recovered) on each cytokine expression level or ventilatory response parameter, general linear models were performed with age, sex, and BMI (Body Mass Index) as covariates (Outcome variable ~ Group + Sex + Age + BMI) using the *glm* function in R. Due to the large number of cytokines evaluated, family-wise Bonferroni p-value adjustments were also implemented. To determine if time post-recovery impacted inflammatory marker expression, general linear models were performed with inflammatory markers as dependent variables and time post-recovery as the predictor variable with age, sex, and/or BMI as additional predictors if they previously showed significant impacts on the current inflammatory marker. The same approach was utilized for the time-post-recovery analysis of ventilatory chemoreflex parameters. In the event of a significant main effect of time post-recovery, pairwise t-tests were performed to determine which time points differed from the control group. Spearman correlations were also performed to determine relationships between cytokine expression and ventilatory control parameters with family-wise p-value corrections via the Holm–Bonferroni method. Normal distributions of each variable were determined via Shapiro-Wilks tests.

## Results

### Participant demographics

Of the 118 participants recruited, 69 were female and 49 were male. Table 2 provides a comparison of demographic factors across control and recovered cohorts. Control and recovered groups had equal distributions of men and women (*p =* 0.89), as well as a similar age (*p =* 0.77) and BMI range (*p =* 0.25). 37 participants in the control group reported being vaccinated against COVID-19 with 2 not vaccinated and 2 who did not disclose vaccination status. Of the recovered group (*n =* 77), 75 participants indicated vaccination against COVID-19 at the time of testing, with some receiving the vaccine before infection and others after recovery. Two recovered participants reported an unvaccinated status. Of the 77 recovered participants, 3 participants are missing ventilatory control data due to voluntarily ending the test early (*n =* 1) or data loss due to a computer error (*n =* 2).

**Table 2:** Participant characteristics

	Female (*n =* 69)		Male (*n =* 49)	
	Control (*n =* 21)	Recovered (*n =* 48)	Control (*n =* 20)	Recovered (*n =* 29)
Age (years)				
Mean ± SD	30.7 ± 10.8	28.6 ± 12.3	26.2 ± 5.1	29.4 ± 10.7
Median	26	24.5	26	26
Weight (lbs)				
Mean ± SD	158.3 ± 37.4	152.3 ± 39.6	200.8 ± 59.0	196.1 ± 54.1
Median	145	141	182	187.5
Height (cm)				
Mean ± SD	162.3 ± 4.8	161.2 ± 6.9	177.5 ± 7.4	176.3 ± 8.4
Median	161.5	161.3	176	177.5
BMI (kg/m^2^)				
Mean ± SD	27.0 ± 5.2	26.2 ± 6.4	28.7 ± 7.1	28.5 ± 6.8
Median	25.6	26	26.8	26.4
SBP (mmHg)				
Mean ± SD	120.5 ± 12.0	119.3 ± 11.9	127.8 ± 10.4	131.9 ± 21.1
Median	118	118	126.5	125
DBP (mmHg)				
Mean ± SD	76.0 ± 8.4	73.1 ± 12.5	77.1 ± 13.5	81.4 ± 13.0
Median	75	74.5	75	79

Physiological measures are reported separated by group and biological sex. There were no significant differences in any factor across sex groups or study groups (control vs. recovered)

*SBP* Systolic blood pressure, *DBP* Diastolic blood pressure

### Inflammatory marker analysis

**Fig. 3:**
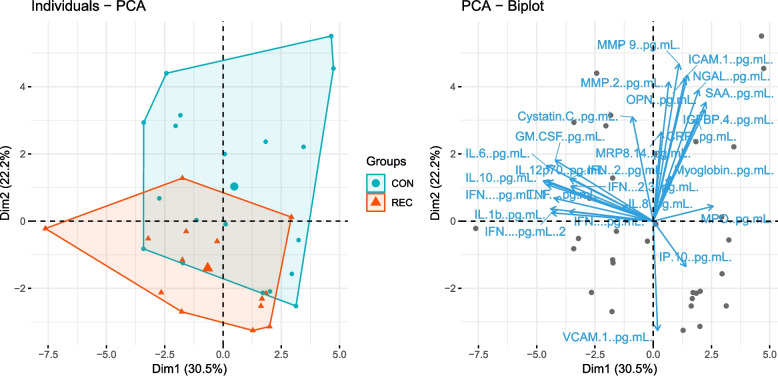
Principal component analysis of inflammatory profiles across control and recovered groups. **A** PCA plot with individual participants represented by points, separated by group (control = blue, recovered = orange). **B** PCA plot with strength and directionality of effect for each inflammatory marker

We first examined if plasma inflammatory marker expression changed after recovery from COVID-19. A principal component analysis (PCA) demonstrated separation of control and recovered groups by inflammatory marker profiles alone (Fig. 3). We found that Myoglobin, NGAL, MMP-2, OPN, IGFBP-4, Cystatin C, and IL-10 all significantly decreased in the recovered group compared to control (Table 3, Figure S1). In addition to these group effects, we also observed a main effect of sex on IGFBP-4 with increased expression in females, and IL-8 with decreased expression in males. We also observed a main effect of BMI on MRP8/14, NGAL, CRP, ICAM-1, MMP-9, all associated with higher expression alongside increased BMI. Finally, lower VCAM-1 was associated with increased BMI (Figure S2).

**Table 3:** Effects of infection and demographic factors on inflammatory marker expression in plasma

*p*-values				
**Vascular Inflammation Panel**	Group	Age	Sex	BMI
Myoglobin	0.0427*	0.307	0.101	0.876
MRP8/14	0.101	0.275	0.150	0.00073***, ł
NGAL	0.00706**	0.669	0.092	0.0057**
CRP	0.161	0.939	0.204	**1.29 × 10**^–5^***, ł
MMP-2	0.00073***, ł	0.675	0.639	0.948
OPN	0.00377**, ł	0.938	0.289	0.704
MPO	0.089	0.550	0.789	0.785
SAA	0.965	0.109	0.521	0.096
IGFBP-4	0.0102*	0.892	0.028*	0.221
ICAM-1	0.327	0.547	0.444	0.0044**
VCAM-1	0.153	0.127	0.153	0.0032**, ł
MMP-9	0.427	0.528	0.490	0.0057**
Cystatin C	0.000549***, ł	0.813	0.948	0.123
**Human Anti-Inflammatory Panel**	**Group**	**Age**	**Sex**	**BMI**
IL-1β	0.467	0.287	0.249	0.135
IL-6	0.174	0.332	0.547	0.688
TNF-α	0.141	0.583	0.544	0.102
IP-10	0.119	0.114	0.410	0.996
IFN-λ1	0.535	0.208	0.461	0.710
IL-8	0.368	0.121	0.032*	0.794
IL-12p70	0.162	0.947	0.104	0.423
IFN-α2	0.286	0.396	0.399	0.544
IFN-λ2/3	0.068	0.969	0.830	0.676
GM-CSF	0.410	0.504	0.756	0.349
IFN-β	0.303	0.158	0.158	0.078
IL-10	0.0499*	0.932	0.827	0.624
IFN-γ	0.620	0.418	0.415	0.190

^*^Indicates significant main effects of each factor at the *p <* 0.05 level, ** indicates *p <* 0.01, *** indicates *p <* 0.001, and ł indicates significant main effects after family-wise (within-panel) Bonferroni adjustment

**Fig. 4:**
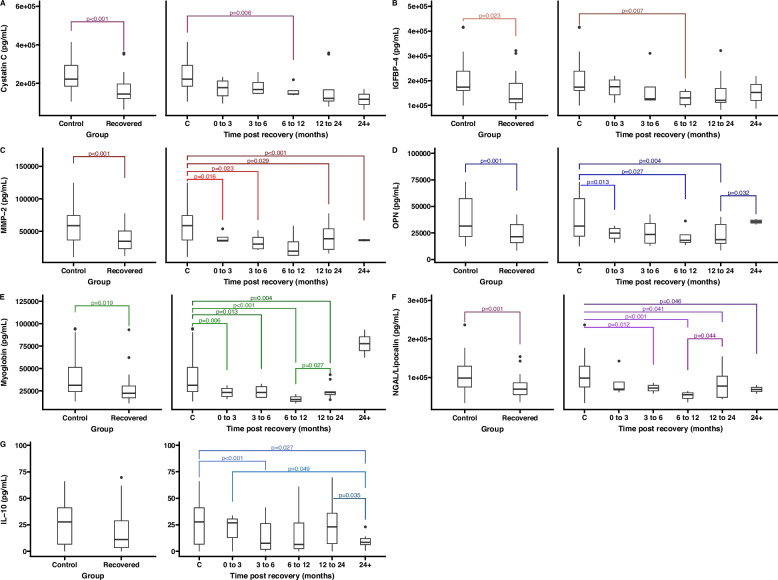
Inflammatory marker expression following COVID-19 recovery. Expression of Cystatin C (**A**), IGFBP-4 (**B**), MMP-2 (**C**), OPN (**D**), Myoglobin (**E**), NGAL/Lipocalin-2 (**F**), and IL-10 (**G**) in control versus all recovered participants (left panels) and at discrete timepoints following recovery from COVID-19 compared to healthy controls (right panels). Tukey style box plots represent medians with boxes representing the first and third quartiles, and whiskers representing the largest value no further than 1.5 * interquartile range (IQR). Significant differences across groups based on post-hoc pairwise t-tests are provided. Versions with individual data points are provided in the supplemental figures

Inflammatory markers were further analyzed to determine if time post-recovery impacted expression levels. The recovered cohort was split into categories based on the amount of time passed since they last tested positive for COVID-19 (*n =* 9 for 0–3 months; *n =* 14 for 3–6, *n =* 13 for 6–12 months, *n =* 14 for 12–24, and *n =* 9 for 24 + months) (Fig. 4). In this analysis, Cystatin C, IGFBP-4, MMP-2, OPN, Myoglobin, NGAL/Lipocalin-2, and IL-10 showed significant impacts of time post-recovery. Contrary to our expectations, several markers of vascular and systemic inflammation showed reduced expression compared to healthy controls as a function of time post-recovery (Figure S3), with some factors returning to baseline levels following 2 years post recovery.

### Baseline lung function and breathing patterns

To determine if lung function was significantly impacted after recovery from COVID-19, participants completed standard spirometry testing. There was no significant difference in airway resistance, noted as FEV_1_/FVC (C: 83.35 ± 6.49%, R: 84.27 ± 7.44%, *p =* 0.54) or FVC (C: 3.68 ± 0.99L, R: 3.38 ± 0.78L, *p =* 0.099) between recovered and control cohorts. This data indicates a return to normal lung function after recovery from infection. We also found no changes in resting ETPCO_2_ (C: 33.67 ± 5.03 mmHg, R: 36.49 ± 4.93 mmHg, *p =* 0.89), SpO_2_ (C: 95.77 ± 1.88%, R: 96.04 ± 1.73%, *p =* 0.31), tidal volume, V_t_ (C: 1.06 ± 0.30L, R: 1.02 ± 0.29L, *p =* 0.26), breathing frequency, V̇_E_ (C: 14.45 ± 3.85 L/min, R: 14.99 ± 4.75 L/min, *p =* 0.44), and resting ventilation, V̇_f_ (C: 14.52 ± 3.45 breaths/min, R: 14.49 ± 3.35 breaths/min, *p =* 0.69) across healthy controls and recovered participants (Figure S4). Resting ETPCO_2_ levels were slightly below the normal range due to the use of a mouthpiece which often leads to moderately increased tidal volumes.

### Ventilatory chemoreflexes

To determine if COVID-19 infection significantly impacted ventilatory chemoreflex sensitivity, we measured the HVR and HCVR, as well as the VRT under hyperoxic and hypoxic oxygen tensions using the Duffin modified rebreathing technique. As expected, we observed that VRT_hypoxia_ was lower than VRT_hyperoxia_, and HCVR_hypoxia_ was higher than HCVR_hyperoxia_ in both recovered and control cohorts. This effect was expected due to interactions between central and peripheral chemoreceptor inputs, and the values within hypoxic and hyperoxic treatments are within the expected ranges.

**Fig. 5:**
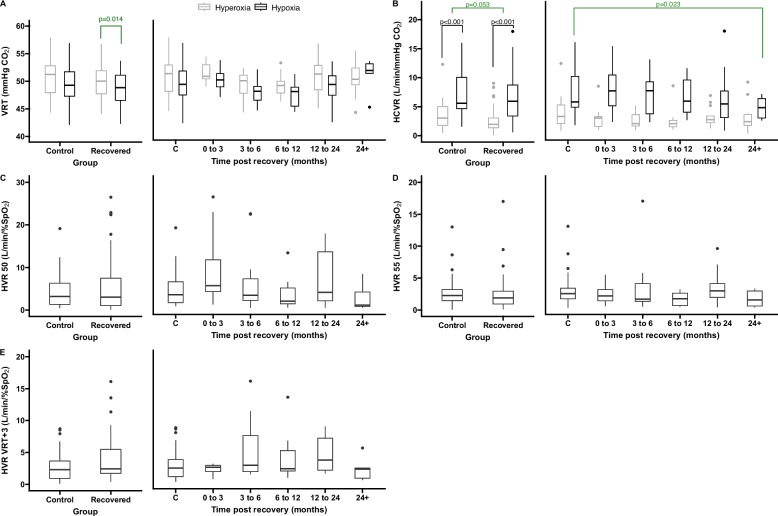
VRT, HCVR, and HVR levels following recovery from COVID-19. Measurements of VRT (**A**), HCVR (**B**), HVR taken at ETPCO_2_ 50 mmHg (**C**), HVR taken at ETPCO_2_ 55 mmHg (**D**) HVR taken at 3 points above VRT (**E**) all at discrete timepoints following recovery from COVID-19 compared to healthy controls. Error bars represent 95% confidence intervals. Significant differences across groups based on post-hoc pairwise t-tests are provided

Our primary comparison of interest was the effect of control versus recovered group on HCVR, HVR, and VRT values within oxygen treatments. We found a near significant main effect of group on HCVR (*p =* 0.053, Fig. 5, Figure S5), with higher mean values in the control group for both HCVR_hyperoxia_ (control mean: 3.35 ± 2.47, CV: 73.57%; recovered mean: 2.60 ± 1.83, CV: 70.40%) and HCVR_hypoxia_ (control mean: 7.83 ± 6.37, CV: 81.38%; recovered mean: 6.05 ± 3.86, CV: 63.80%), although these differences were not significant via post-hoc comparisons (*p =* 0.134 and *p =* 0.167, respectively). We also identified main effects of age (*p =* 0.014; within tests: HCVR_hyperoxia_ *p =* 0.001, rho = 0.31; HCVR_hypoxia_ *p =* 0.003, rho = 0.30) and sex (*p =* 0.034) on the HCVR. The effect of sex on the HCVR was driven by a higher HCVR_hypoxia_ in men (men: 8.3 L/min/mmHg CO_2_, women: 5.8 L/min/mmHg CO_2_, *p =* 0.021). As expected, HCVR sensitivity was always higher with co-stimulus with hypoxia (*p <* 0.001).

There was no effect of group on the VRT (hyperoxia control mean: 50.56 ± 3.62, CV: 7.15%; hyperoxia recovered mean: 49.66 ± 2.94, CV: 5.92%; hypoxia control mean: 49.51 ± 3.15, CV: 6.38%; hypoxia recovered mean: 48.80 ± 2.86, CV 5.85%, *p =* 0.505). However, we did find a significant main effect of sex (*p <* 0.001) and BMI (*p =* 0.029) on this measure. The effect of sex was driven by both higher VRT_hyperoxia_ (men: 51.6 mmHg CO_2_, women: 49.2 mmHg CO2, *p <* 0.001) and VRT_hypoxia_ (men:50.8 mmHg CO2, women: 47.7 mmHg CO2, *p <* 0.001) in men. Higher BMI was associated with higher VRT_hyperoxia_ (*p =* 0.016, rho = 0.26) and VRT_hypoxia_ (*p =* 0.003, rho = 0.31).

There was no main effect of group on any of the HVR measures (HVR_50_: control mean: 5.21 ± 6.59, CV: 126.43%; recovered mean: 5.24 ± 6.42, CV: 122.55%; HVR_55_: control mean: 3.07 ± 2.92, CV: 95.15%; recovered mean: 2.45 ± 2.85, CV: 116.65%; HVR_VRT+3_: control mean: 2.85 ± 2.58, CV: 90.63%; recovered mean: 3.68 ± 3.50, CV: 95.04%), although there was a main effect of sex on HVR_50,_ with a higher mean in men (Men: 7.8 L/min/SpO_2_, women: 3.9 L/min/SpO_2_, *p =* 0.010).

Because of the observable trend towards an overall decreased HCVR and VRT across oxygen tensions and in both study cohorts, we performed a more in-depth analysis and separated the recovered cohort into time post recovery groups. Of the 74 recovered participants, 11 participants did not provide confident estimates on when they tested positive for COVID-19, resulting in a sample size of *n =* 9 for 0–3 months; *n =* 16 for 3–6 months; *n =* 13 for 6–12 months; *n =* 15 for 12–24 months; and *n =* 10 for 24 + months. There was a main effect of time post recovery for the HCVR, driven by a significant decrease in the recovered group at 24 + months compared to the control group (*p =* 0.020). There were no significant differences in any HVR or VRT measure compared to the control group at any timepoint of recovery.

### Cytokine expression and ventilatory chemoreflexes

**Fig. 6:**
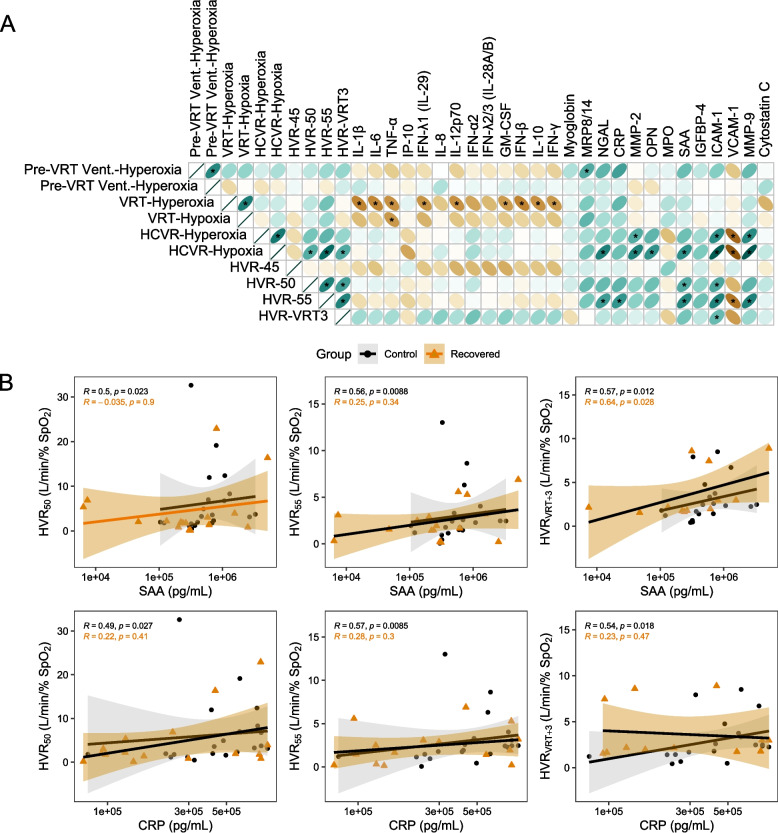
Correlation analysis of candidate ventilatory control parameters and inflammatory marker expression. **A** A matrix visualizing Spearman rank correlations of all inflammatory markers versus multiple ventilatory control parameters. Color represents the directionality and strength of the correlation coefficient. Asterisks represent significant correlations after family-wise multiple comparisons corrections. **B** Raw data plots representing correlations between candidate inflammatory markers SAA and CRP with each HVR measurement. Data are presented on a log10 scale, with point shapes and colors representing measures within control and recovered groups. A linear fit is included with standard error indicated by the shaded region

Finally, we conducted a correlation analysis to determine if systemic inflammatory marker expression was associated with any of the ventilatory chemoreflex measurements. Several significant associations were identified (Fig. 6, Figure S6). Among these, CRP, SAA, and ICAM-1 were consistently associated with HVR_50_ and HVR_55_ after multiple comparisons corrections (Fig. 6A). SAA, ICAM-1 and other vascular inflammation markers were also associated with HCVR_hypoxia_. Higher levels of these specific inflammatory cytokines were generally associated with higher HVRs. Also of note, many of the anti-viral response markers were negatively associated with the VRT. A lower VRT results in higher ventilation at a given PCO_2_ level above the VRT.

### Within subject findings

**Fig. 7:**
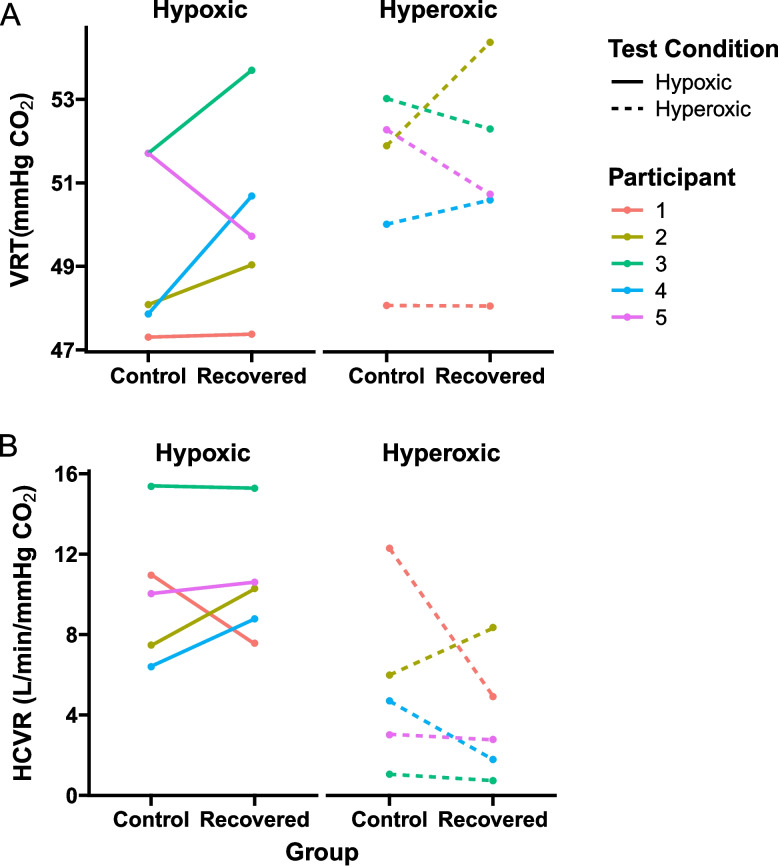
Within subject comparison of VRT and HCVR. Within subject comparisons of (**A**), VRT and (**B**), HCVR show differences in the control of breathing parameters pre- and post-COVID infection. Comparisons are made within both hyperoxic and hypoxic oxygen tensions

From the control study cohort, five participants returned after COVID-19 infection to participate in the study a second time as a recovered participant, allowing valuable, but underpowered, paired pre- and post-COVID measures (Fig. 7; Table S1). A two-way repeated measures ANOVA revealed no significant main effects of control versus recovered timepoints for the HCVR (timepoint *p =* 0.652, test *p =* 0.082, interaction *p =* 0.105), or VRT (timepoint: *p =* 0.513, test: *p =* 0.157, interaction: *p =* 0.489).

## Discussion

### Inflammatory biomarkers following COVID-19 recovery

The primary aim of this study was to determine if COVID-19 led to prolonged changes in systemic inflammatory profiles and if these changes were linked to modifications in the chemoreflex control of breathing. Our findings of decreased plasma levels of many vascular inflammatory cytokines after recovery from COVID-19 compared to a healthy control group may indirectly support previous findings of persistent immune dysfunction following acute infection [27]. This is consistent with the findings of Sabioni et al. (2023) who also observed decreases in pro- and anti-inflammatory serum cytokine and chemokine markers following 12–15 months post-COVID-19 recovery [28], and therefore confirming the rebound effect in which expression of these vascular inflammation mediators becomes blunted following recovery in response to significant overexpression during the acute COVID-19 infection. Indeed, in a prior study by our group, we found many of these inflammatory factors to be upregulated during acute COVID-19 infection [19]. Additionally, reduced circulating levels may indicate ongoing COVID-related pathology in which these inflammatory markers are actively recruited to tissue, such as at sites of vascular injury, rather than circulating in peripheral venous blood. Similarly, the pathways responsible for clearing inflammatory markers may be upregulated during the recovery period, subsequently resulting in decreased expression of these inflammatory markers following recovery. However, aside from these observations and the findings of Sabioni et al. (2023) [28], we are not aware of any other studies demonstrating this reduction in inflammatory markers below baseline during recovery, and such studies which follow biomarker expression in participants for several months after recovery from acute illnesses are rare.

In addition to the effects of COVID-19 itself, we also found main effects of sex and BMI in some inflammatory biomarkers These findings were expected, considering that sex hormones play a key role in altering inflammatory cytokine expression [29] and increased expression of pro-inflammatory cytokines in those with higher BMI is often reported [30]. Indeed, the higher levels of IGFBP-4 and IL-8 we observed in women compared to men have been identified in some prior work [31], although other studies report no sex-based differences in baseline plasma levels of these markers across sex [32–35]. More commonly, differences in the amplitude of expression of cytokines in response to stimuli across sex groups are reported. Furthermore, previous studies demonstrate that NGAL/Lipocalin-2 expression changes are linked to obesity and higher BMI [36].

### Baseline lung function and breathing patterns

In this cohort study, we found that lung function measured by spirometry, as well as baseline breathing patterns, were not significantly impacted following COVID-19. However, our cohort included individuals with mild to moderate COVID-19, with only two participants reporting that they sought care at a hospital. Therefore, these data are consistent with prior findings that airway resistance, lung volume, and diffusion capacity return to baseline within one year of recovery in mild to moderate cases not requiring hospitalization [37]. In contrast, many studies examining individuals with moderate to critical disease, including hospitalized patients, indicate significant impairment in lung function months after hospital discharge or recovery [38–41]. The age of our study cohort is also lower than some other studies, perhaps indicating that long-term impacts of COVID-19 on lung function are more severe in older populations. Indeed, Lewis et al. (2021) indicated that age was independently associated with prolonged impaired lung function following recovery from mild to moderate COVID-19 symptoms [37].

### Ventilatory chemoreflexes

We observed small but significant decreases in the HCVR_hypoxia_ several months after recovery. The functional significance of a deceased HCVR is that for a given increase in arterial PCO_2_, there is a lower increase in ventilation. The trend toward a reduction in VRT_hypoxia_ may be a compensatory response to the reduced HCVR_hypoxia_ to maintain normal minute ventilation at a given arterial PCO_2_. Reduced CO_2_ chemosensitivity may influence sleep disordered breathing pathology in complex ways, such as prolonging apnea or delaying arousal in obstructive sleep apnea. However, ventilatory control measures collected during wakefulness may not represent ventilatory control during sleep.

There were no significant differences in any of the HVR measures across study cohorts. However, across the complete study populations, higher levels of acute-phase inflammatory markers CRP and SAA were associated with higher HVRs. This is consistent with prior work demonstrating that acute-phase inflammatory signaling plays a key role in hypoxic chemosensitivity and ventilatory acclimatization to hypoxia. However, this particular association has not yet been reported. It is important to note that the relationship between systemic inflammation and changes in the chemoreflex control of breathing depends on the time domain of exposure, with chronic systemic inflammation being associated with potential blunting of hypoxic chemosensitivity, as observed in chronic obstructive pulmonary disease (COPD) or emphysema [7, 42]. Overall, more work is needed to determine links between systemic inflammation and neural control of breathing.

Ventilatory chemoreflex sensitivity to both hypoxia and hypercapnia are highly variable across individuals and show remarkable plasticity within individuals as a result of environmental exposures, such as travel to high altitude, or pathology, such as sleep apnea, chronic obstructive lung disease, or heart failure [43]. Due to this high degree of variability, it may be difficult to observe significant changes in these reflexes across a diverse population following recovery from an acute illness. While this challenge highlights the value of our current findings, it also highlights the need for future studies which can target within-subject changes in these reflexes throughout the development of different pathologies, although such studies are challenging.

### Limitations

While our study provides important insight into the impact of acute infection on systemic inflammation and control of breathing, it has some noteworthy limitations. Due to the cross-sectional study design, we cannot rule out that differences observed across control and recovered groups were not the result of other factors which may have differed systematically across the two populations. However, it is challenging to capture the same individuals before and after COVID-19 infection for a paired study design. Nonetheless, we carefully aimed for matched cohorts and, when possible, re-recruited participants from the control group after they had contracted COVID-19. This study did not use complete participant medical records; thus, we depended on participant reporting of comorbidities which may have influenced their baseline systemic inflammatory status. Participants were also not fasting at the time of blood sample collection due to comparison of these samples with unfasted ICU blood samples in a related study [19]. However, due to the large sample size and heterogeneous distribution of samples across daily timepoints between both treatment groups, our inflammatory marker levels should not differ across groups based on recent diet. We also cannot validate if participants in the control population had experienced an asymptomatic COVID-19 infection. Finally, we are underpowered to determine any significant impacts of vaccination status on these outcomes since only a few participants reported an unvaccinated status.

## Conclusion

Our findings demonstrate that several key pro- and anti-inflammatory cytokines are reduced in circulation following recovery from COVID-19. We also show small but physiologically significant decreases in the VRT_hypoxia_ and HCVR_hypoxia_ during recovery which may be linked to prior large-scale sustained systemic inflammation during the COVID-19 cytokine storm. Taken together, these data suggest that an acute viral infection such as COVID-19 has long-term impacts on inflammatory status and the chemoreflex control of breathing. These changes in breathing reflexes may also be related to respiratory symptoms, such as persistent dyspnea [44] and increased sleep disordered breathing following recovery; however, these links warrant further study.

## Supplementary Information

Supplementary Material 1.

## Data Availability

All data and materials generated for this study are available under the “Supplemental Material” section of this article.

## Abbreviations

COVID-19: Coronavirus Disease of 2019
IL-6: Interleukin-6
IL-1β: Interleukin-1beta
TNF-α: Tumor necrosis factor alpha
HVR: Hypoxic ventilatory response
HCVR: Hypercapnic ventilatory response
VRT: Ventilatory recruitment threshold
UC (Riverside): University of California (Riverside)
ICU: Intensive care unit
mL: Milliliters
EDTA: Ethylenediaminetetraacetic acid
CXCL8 (IL-8): Chemokine (C-X-C motif) ligand 8 (interleukin-8)
IL-10: Interleukin-10
IL-12p70: Interleukin-12, protein subunit 70
IFN-α2: Interferon-alpha2
IFN-β: Interferon-beta
IFN-λ1 (IL-29): Interferon-lambda1 (interleukin-29)
IFN-λ2/3 (IL-28a/b): Interferon-lambda2/3 (interleukin-28 isoform a/isoform b)
IFN-γ: Interferon-gamma
CXCL10: Chemokine (C-X-C motif) ligand 10
IP-10: Interferon gamma-induced protein 10
GM-CSF: Granulocyte–macrophage colony-stimulating factor
MRP8/14: Myeloid-related proteins 8 and 14
NGAL: Neutrophil gelatinase-associated lipocalin
CRP: C-Reactive Protein
MMP-2: Matrix metallopeptidase 2
OPN: Osteopontin
MPO: Myeloperoxidase
SAA: Serum Amyloid A
IGFBP-4: Insulin-like growth factor binding protein 4
ICAM-1 (CD54): Intercellular adhesion molecule 1 (cluster of differentiation 54)
VCAM-1 (CD106): Vascular cell adhesion molecule 1 (cluster of differentiation 106)
MMP-9: Matrix metallopeptidase 9
ECG: Electrocardiogram
BTPS: Body temperature and pressure, saturated
O_2_: Oxygen
CO_2_: Carbon dioxide
PO_2_: Partial pressure of oxygen
PCO_2_: Partial pressure of carbon dioxide
P_ET_CO_2_: End-tidal partial pressure of carbon dioxide
P_ET_O_2_: End-tidal partial pressure of oxygen
P_I_O_2_: Partial pressure of inspired oxygen
mmHg: Millimeters of mercury
SpO_2_: Peripheral oxygen saturation
Tv: Total ventilation
V_i_: Inspiratory volume
V̇_E_: Minute ventilation
V_t_: Tidal volume
V̇_f_: Ventilatory frequency
CV: Coefficient of variation
BMI: Body mass index
SBP: Systolic blood pressure
DBP: Diastolic blood pressure
PCA: Principal component analysis
IQR: Interquartile range
FEV_1_: Forced expired volume in 1s
FVC: Forced vital capacity
COPD: Chronic obstructive pulmonary disease
