# Supplementary material for: Long-term impacts of COVID-19 on systemic inflammation and control of breathing reflexes: an observational cohort study
Source: Respir Res. 2026 Jan 21;27:70. doi: 10.1186/s12931-025-03473-6 (PMC12908332; doi:10.1186/s12931-025-03473-6)
Supplement: Supplementary file 1 — Supplementary Material 1. [file 12931_2025_3473_MOESM1_ESM.pdf]

Long-term impacts of COVID-19 on systemic inflammation and control of breathing reflexes: an observational cohort study

Supplemental Materials

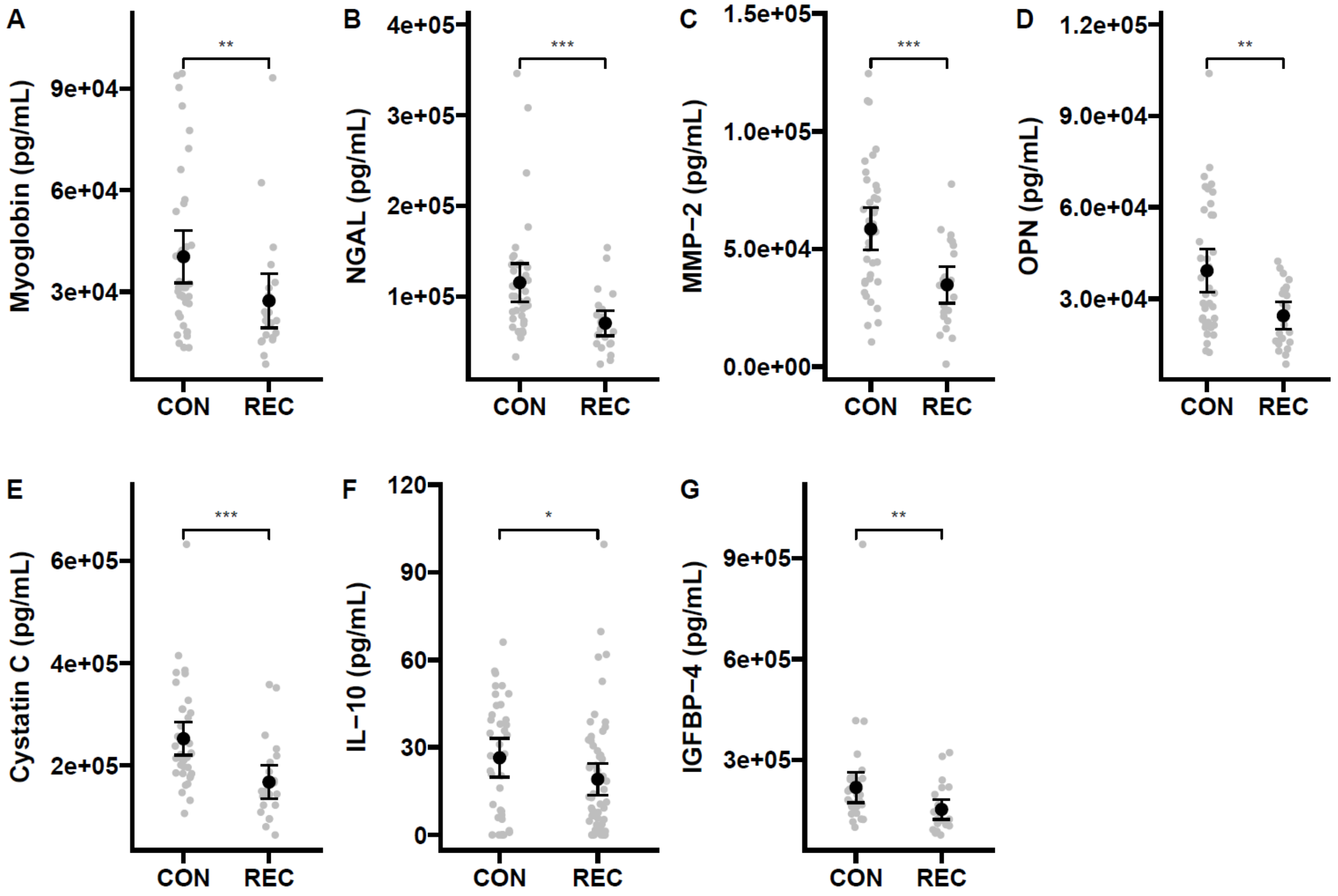

Figure S1. Plasma inflammatory markers across control and COVID-19 recovered groups. Group means are provided with error bars representing 95% confidence intervals. Asterisks represent significant differences at the p <0.05 (*), p<0.01 (**), or p<0.001 (***) level.

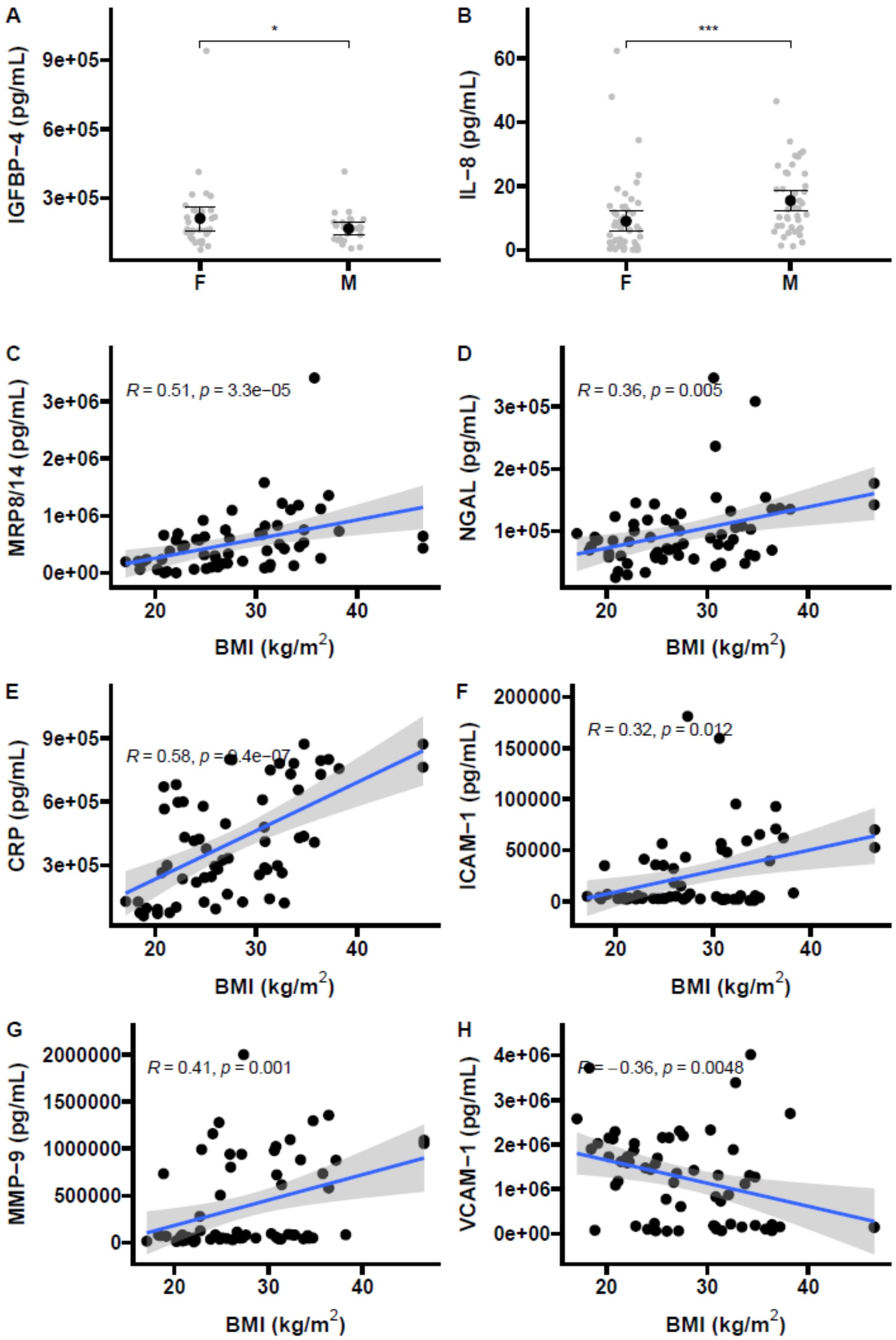

Figure S2. Inflammatory biomarker expression across sex and BMI. (A-B) Inflammatory biomarker expression across men and women. Group means are provided with error bars representing 95% confidence intervals. Asterisks represent significant differences at the p <0.05 (*), p<0.01 (**), or p<0.001 (***) level. **(C-G)** Results of Spearman rank correlations are provided, with linear fits and standard errors represented by blue lines and grey shaded regions.

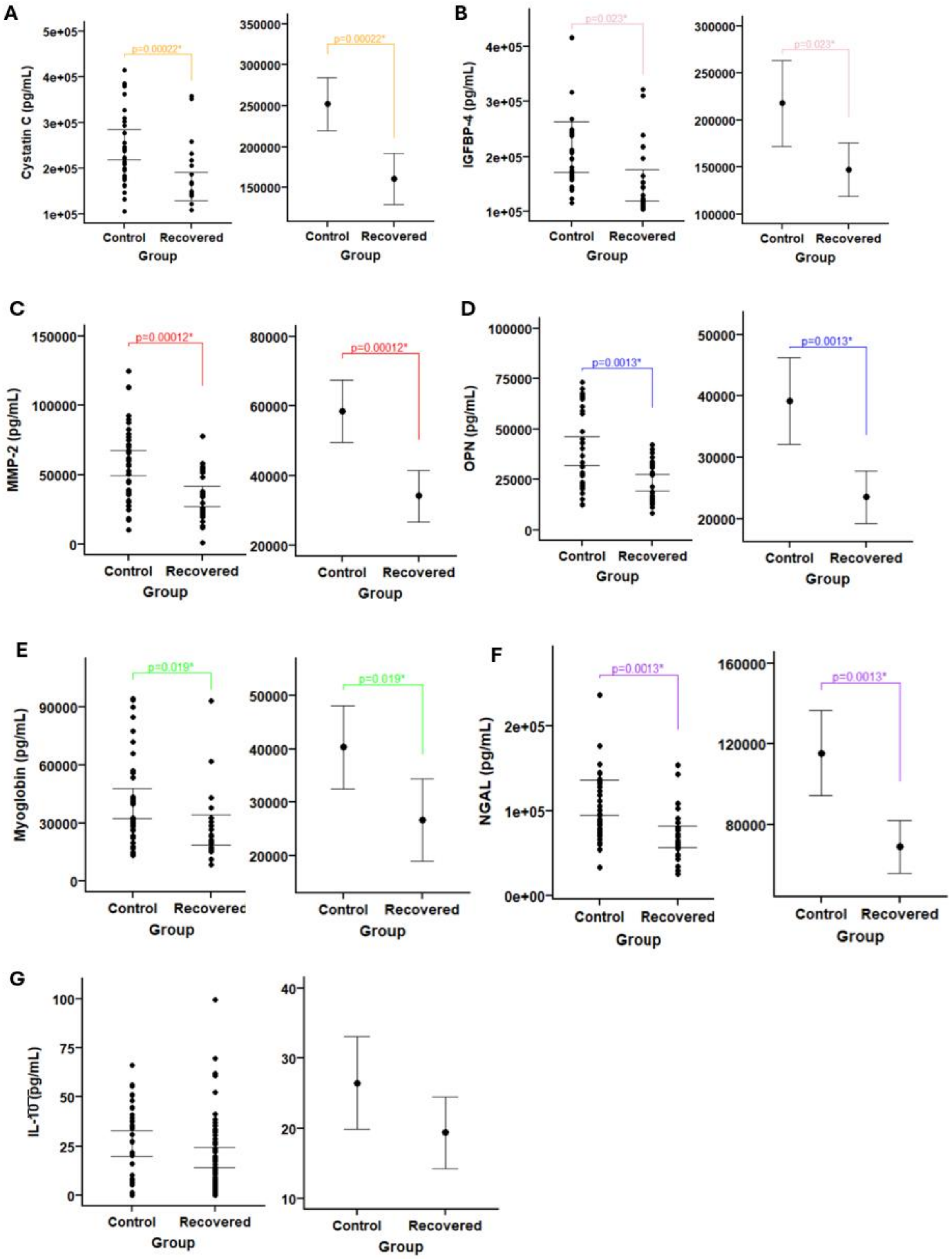

Figure S3. Inflammatory biomarker expression following recovery from COVID-19. Figures indicate the same statistics provided in Figure 4 within the main text, with individual data points provided in each left panel and means with 95% confidence intervals in each right panel.

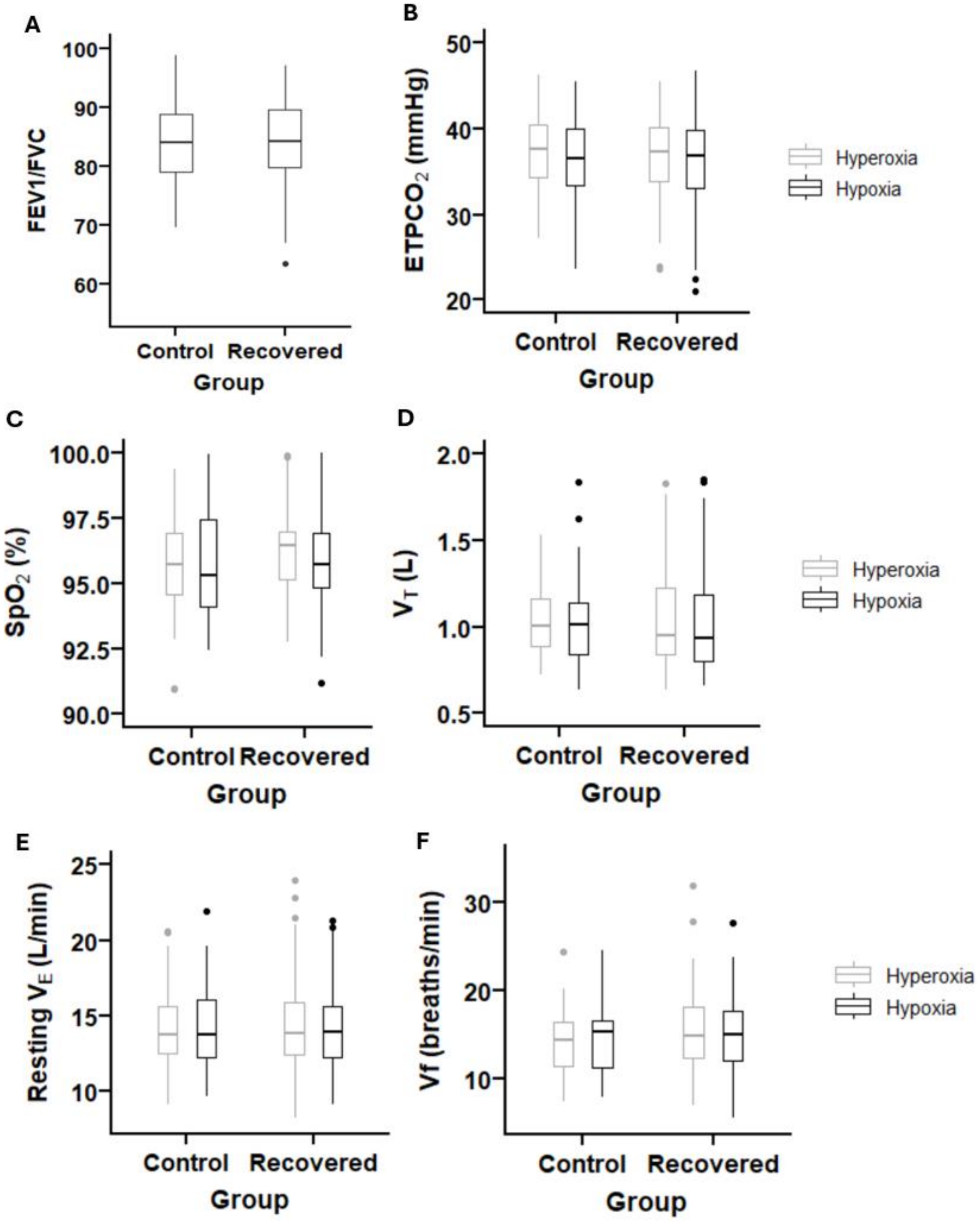

Figure S4. Lung function and resting breathing patterns. Comparisons of FEV_1/FVC (**A**), and resting end-tidal PCO_2 (**B**), SpO_2 (**C**), tidal volume (**D**), breathing frequency (**E**), and minute ventilation (**F**) across control and recovered groups. Tukey style box plots represent medians with boxes representing the first and third quartiles, and whiskers representing the largest value no further than 1.5 * IQR, Significant differences across groups based on post-hoc pairwise t-tests are provided.

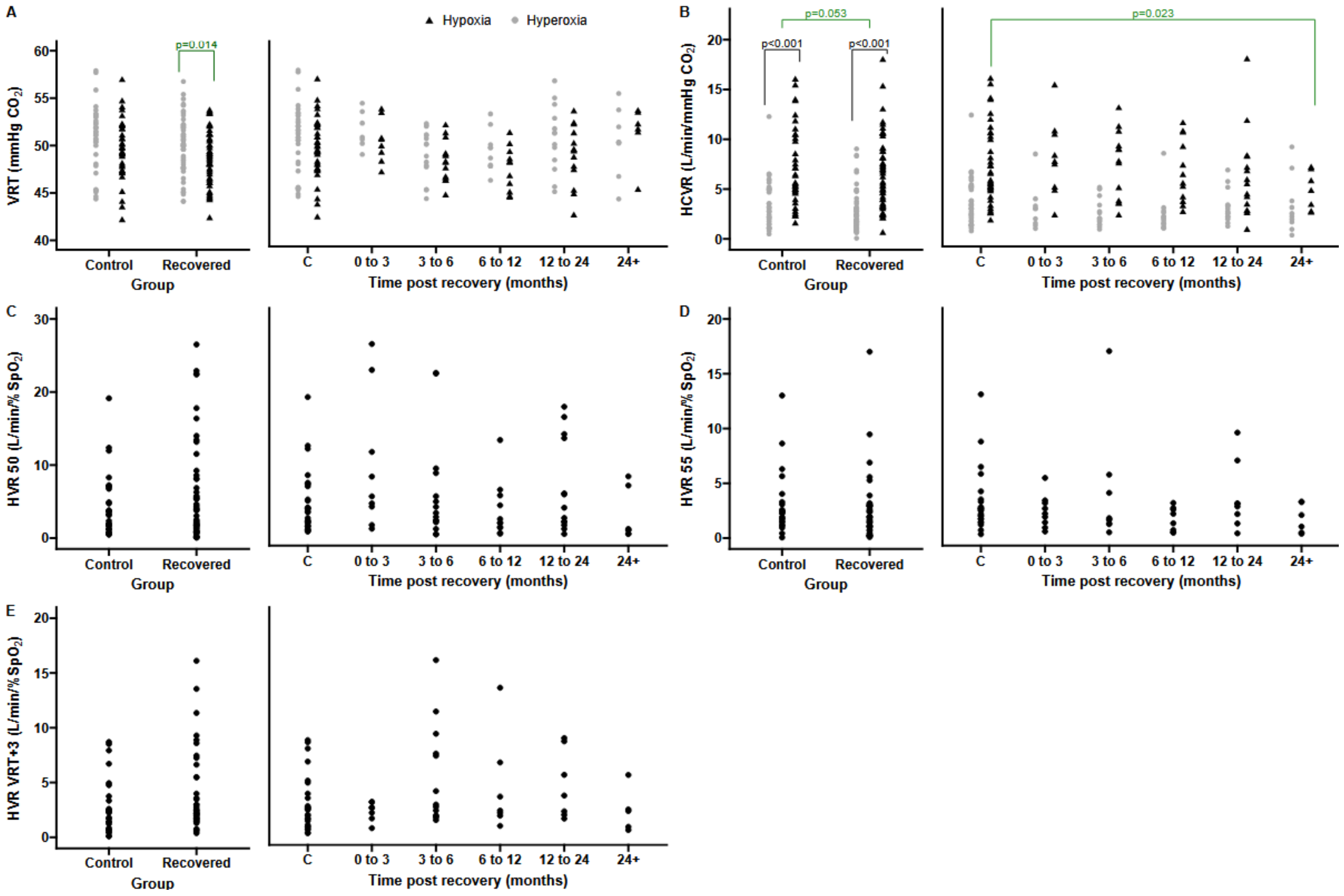

Figure S5. VRT, HCVR, and HVR plots with individual data points. Figures indicate the same statistics provided in figure 5 within the main text, with individual data points.

All participants

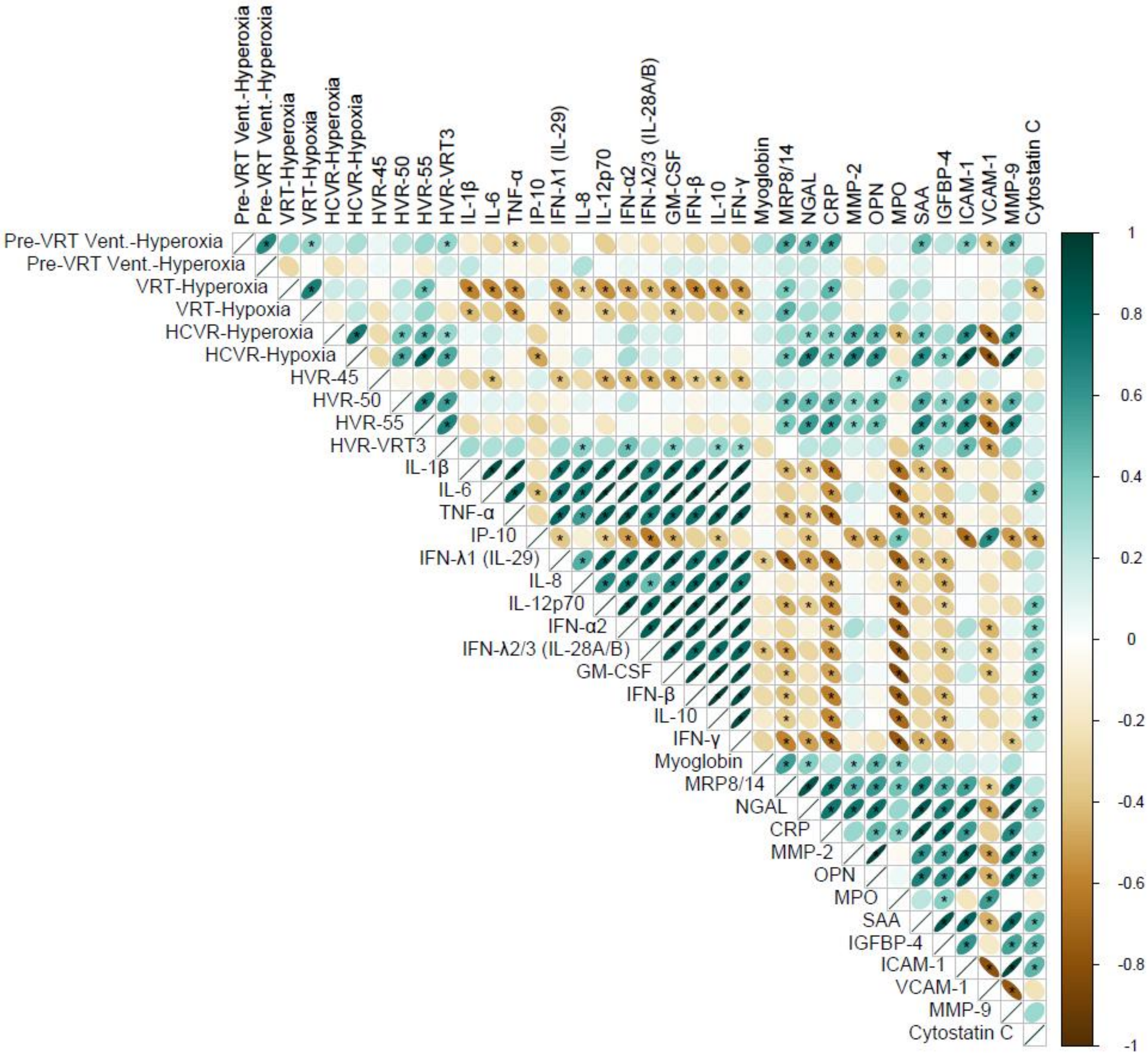

Control cohort

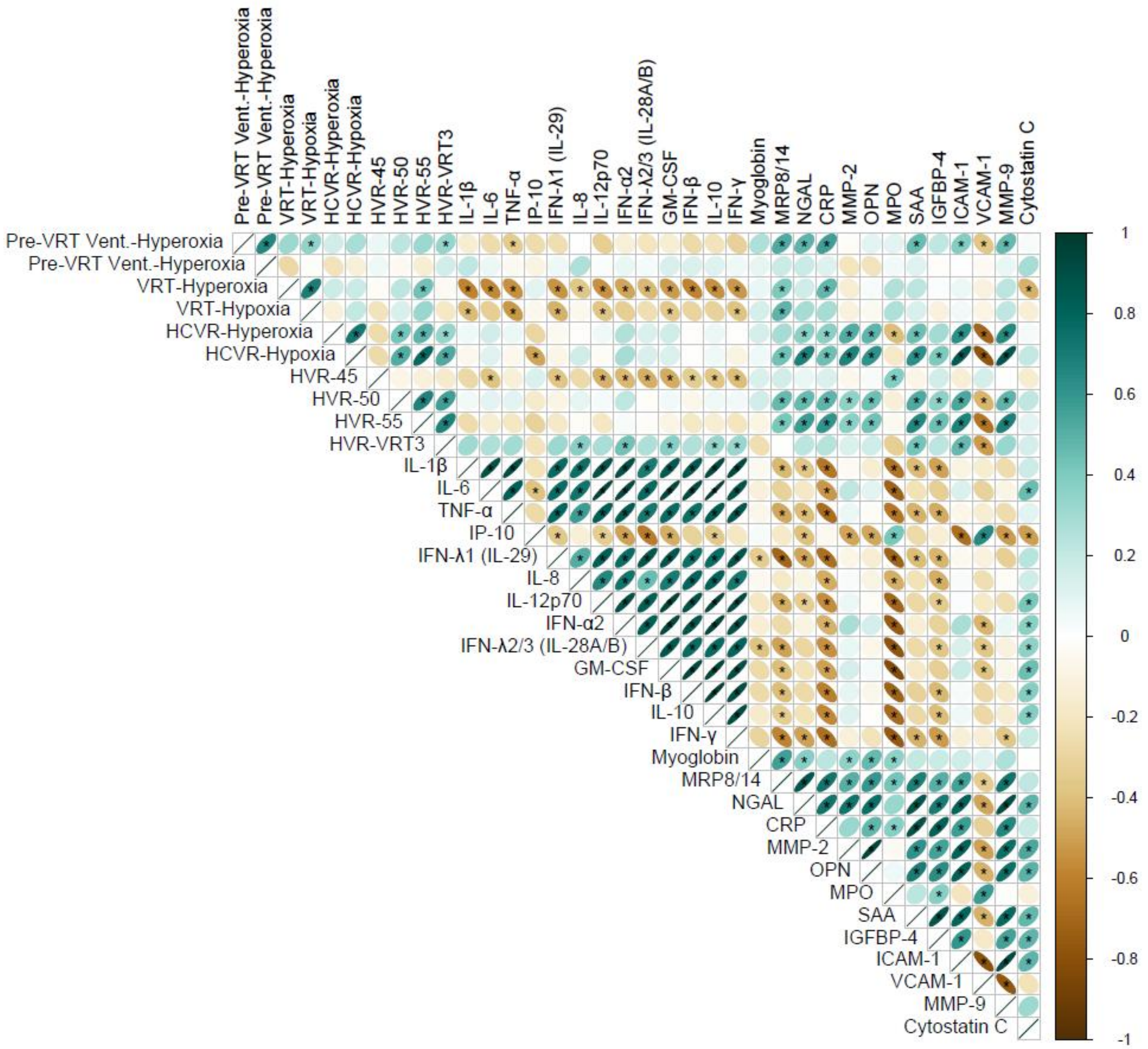

Recovered cohort

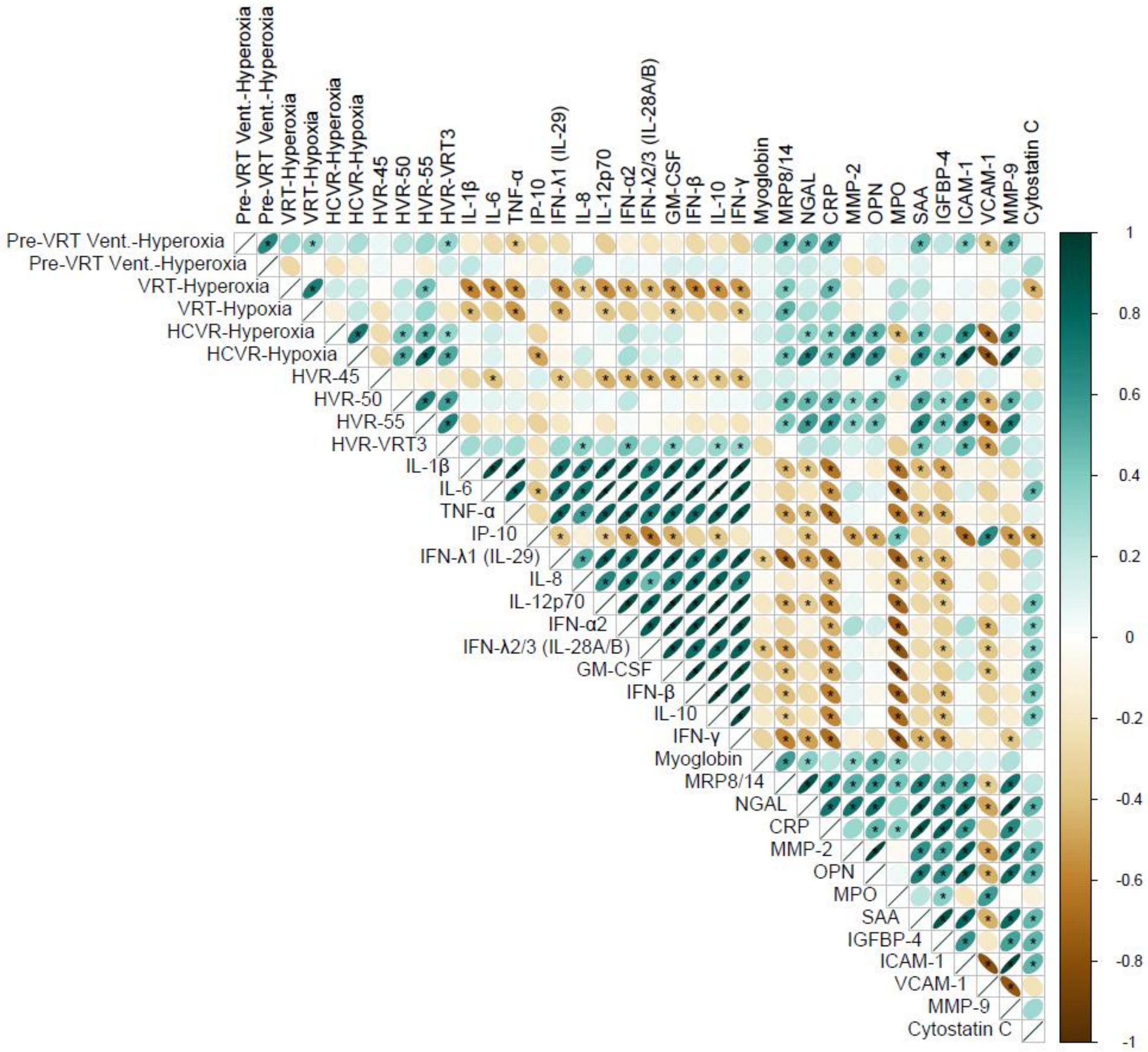

Figure S6. Correlation analysis of candidate ventilatory control parameters and inflammatory marker expression. Matrices visualizing Spearman rank correlations of all inflammatory markers versus multiple ventilatory control parameters. Color represents the directionality and strength of the correlation coefficient. Asterisks represent significant correlations without multiple comparisons corrections. Matrices represent analyses of the full study cohort (recovered and control pooled together) (top/first), the control cohort only (middle/second), and the recovered cohort only (bottom/third).

TABLES

	Group	Test	VRT	HCVR	HVR VRT+3
**Participant 1**	Control	Hyperoxia	48.064	12.290	0.208
		Hypoxia	47.307	10.985	
	Recovered	Hyperoxia	48.050	4.938	6.751
		Hypoxia	47.377	7.538	
**Participant 2**	Control	Hyperoxia	51.886	5.989	1.487
		Hypoxia	48.085	7.453	
	Recovered	Hyperoxia	54.363	8.320	1.487
		Hypoxia	49.036	10.271	
**Participant 3**	Control	Hyperoxia	53.017	1.051	3.312
		Hypoxia	51.707	15.401	
	Recovered	Hyperoxia	52.289	0.736	5.405
		Hypoxia	53.693	15.284	
**Participant 4**	Control	Hyperoxia	50.009	4.692	0.882
		Hypoxia	47.860	6.422	
	Recovered	Hyperoxia	50.588	1.784	7.581
		Hypoxia	50.684	8.777	
**Participant 5**	Control	Hyperoxia	52.270	3.042	5.412
		Hypoxia	51.709	10.039	
	Recovered	Hyperoxia	50.726	2.764	3.232
		Hypoxia	49.720	10.609	

Table S1. Within subject ventilatory parameters. Values are representative of differences in control of breathing between control and recovered same participants. Participants 1 and 4 were tested again 4-6 months post recovery and the remaining participants 0-3 months post recovery.
